# A phase II randomized controlled trial of orally administered yeast-derived β-glucan for alleviating chemoradiotherapy-induced oral mucositis in nasopharyngeal carcinoma patients

**DOI:** 10.3389/fonc.2026.1775554

**Published:** 2026-03-09

**Authors:** Xiwei Xu, Fang Li, Fanglin Xie, Feixia Zhuo, Xiaomin Xie, Qingfeng Yang, Renwei Jiang, Jinman Chen, Siyang Wang, Yuanling Luo, Lingling Yu, Zongping Han

**Affiliations:** 1 Department of Radiation Oncology, The Fifth Affiliated Hospital, Sun Yat-sen University, Zhuhai, Guangdong, China; 2 Department of Clinical Nutrition, The Fifth Affiliated Hospital, Sun Yat-sen University, Zhuhai, Guangdong, China; 3 Nasopharyngeal Cancer Prevention and Treatment Center, The Fifth Affiliated Hospital of Sun Yat-sen University, Zhuhai, Guangdong, China

**Keywords:** chemoradiotherapy, nasopharyngeal carcinoma, nutritional status, oral mucositis, randomized controlled trial, supportive care, yeast-derived β-glucan

## Abstract

**Background:**

Patients with locoregionally advanced nasopharyngeal carcinoma (NPC) undergoing chemoradiotherapy frequently experience severe oral mucositis, with incidence rates ranging from 78.6%-88%. This adverse effect often disrupts therapeutic adherence and negatively impacts nutritional intake. This Phase II trial evaluated oral yeast-derived β-glucan (PGG) for alleviating mucositis and improving nutrition in NPC patients.

**Methods:**

Sixty-three stage III-IVa NPC patients receiving radical radiotherapy (70 Gy/33F) with concurrent cisplatin were randomized to PGG supplementation (Experimental group, 5 g/10kg/day, n=30) plus routine care or routine care alone (Control group, n=30). Mucositis severity (RTOG criteria), nutritional parameters (PG-SGA, body composition), and hematological indices were assessed weekly.

**Results:**

The experimental Group showed significantly reduced mucositis severity: 70% achieved grade 0-I (vs. 36.7% controls; U = 266.000, p=0.004), with grade III incidence at 6.67% (vs. 26.67%). Nutritional outcomes improved in the experimental Group, evidenced by lower PG-SGA scores at week 4 (10.93 ± 2.60 vs. 12.37 ± 2.39, p=0.03), attenuated weight loss during weeks 3-4 (p<0.05), and increased body fat percentage (p<0.05). No intergroup differences occurred in pain scores, muscle mass, or hematological parameters (leukocytes, hemoglobin, platelets, lymphocyte subsets).

**Conclusion:**

Oral PGG significantly reduces severe mucositis incidence and mitigates nutritional deterioration during NPC chemoradiotherapy without added toxicity.

## Introduction

Nasopharyngeal carcinoma (NPC), a head and neck malignancy highly prevalent in southern China, presents as locally advanced (stage III–IVa) in approximately 60%–70% of patients at initial diagnosis (1). The current standard treatment regimen consists of radical intensity-modulated radiotherapy (IMRT) combined with cisplatin-based concurrent chemotherapy. Although this strategy significantly improves locoregional control rates and survival outcomes (2, 3), it is associated with a high incidence (78.6%–88%) of radiation-induced oral mucositis (RTOM) (4, 5). RTOM is characterized by progressive epithelial barrier disruption and ulcer formation, typically manifesting when the cumulative radiation dose reaches ≥30 Gy (around week 3) and persisting for at least two weeks post-treatment (6, 7). Severe mucositis (Grade III/IV) not only causes intense pain, dysphagia, and a sharp decline in nutritional intake but can also lead to secondary infections, treatment interruptions, and severely compromised quality of life, making it a key dose-limiting toxicity that constrains treatment efficacy (8, 9). Despite the routine clinical implementation of stepwise management (e.g., topical analgesics, mucosal protectants, anti-infectives, and nutritional support), the control rate for moderate-to-severe mucositis remains suboptimal, highlighting an urgent need for novel intervention strategies that are both effective and safe (10–13).

Yeast-derived β-glucan (PGG), a natural polysaccharide dietary fiber, exerts multifaceted regulatory roles in tissue repair. This bioactive compound stimulates Dectin-1 receptors expressed by macrophages, while simultaneously inducing fibroblast multiplication and enhancing collagen production (14–16). Preclinical studies have demonstrated that PGG accelerates epithelial regeneration and angiogenesis, with its mechanism involving the restoration of inflammatory homeostasis mediated by the NF-κB signaling pathway (17, 18). In chronic wound models like diabetic foot ulcers, topical application of PGG achieved wound healing in 40%–45% of patients, showing significantly superior efficacy compared to growth factor gels (19, 20). However, its potential in the field of preventing chemoradiation-induced mucosal injury has not been systematically evaluated, and clinical evidence is particularly lacking for high-mucositis-toxicity tumors like NPC undergoing concurrent chemoradiotherapy.

Based on the above background, this study aims to conduct a phase II randomized controlled clinical trial. It will systematically evaluate the efficacy and safety of oral PGG in alleviating radiation-induced RTOM and improving nutritional status in patients with locoregionally advanced NPC receiving concurrent chemoradiotherapy. The goal is to provide NPC patients with a novel and effective adjunctive therapy, thereby optimizing clinical management strategies, alleviating patient suffering, enhancing quality of life and treatment adherence, and ultimately improving patient prognosis.

## Materials and methods

### Patient screening

This Phase II clinical investigation (Registration No.: MR-44-23-005129; Registered via https://www.medicalresearch.org.cn/) employed a prospective, randomized, controlled design at a single institution. Between March and December 2023, we recruited consecutive treatment-naïve patients presenting with locoregionally advanced nasopharyngeal carcinoma at our institution. Eligibility requirements included: ① age 18–80 years; ② histologically verified stage III-IVA disease per AJCC 8th edition criteria; ③ planned definitive chemoradiotherapy regimen; ④ Karnofsky Performance Status (KPS) score of 70 or above; ⑤ willingness to provide written informed consent. Exclusion parameters comprised: ① pregnancy or breastfeeding status; ② poorly managed comorbidities (including HbA1c exceeding 8%, sustained systolic hypertension >160 mmHg, or NYHA Class III-IV cardiac impairment); ③ patients with autoimmune diseases or immunodeficiency; ④ patients who received immunomodulatory therapy within 4 weeks prior to enrollment; ⑤ patients deemed ineligible for participation by the investigators. As this was an exploratory Phase II randomized trial, the target sample size was primarily based on feasibility (anticipated recruitment over the study period and available resources) and to obtain preliminary estimates of efficacy and safety for planning a subsequent confirmatory trial; therefore, no formal *a priori* power calculation was performed. Eligible participants were assigned in a 1:1 ratio to the PGG group or the control group using a computer-generated randomization sequence. Allocation was concealed by an independent coordinator who was not involved in outcome assessment or statistical analysis.

### Grouping and treatment protocol

The treatment protocol for all participants involved standardized chemoradiation therapy. Radiation delivery was accomplished using either Elekta Infinity/Synergy linear accelerators or TomoTherapy Radixact X7 platforms for intensity-modulated radiation treatment (IMRT). Dose prescriptions followed established protocols: the primary nasopharyngeal tumor and involved lymph nodes received 70 Gy administered in 33 fractions, while high-risk target volumes (CTV1) were treated with 60 Gy across the same fractionation schedule. Lower-risk regions (CTV2) were irradiated with 54 Gy delivered in 33 treatment sessions. All treatment plans underwent dose verification (γ passing rate >95% at 3%/3 mm criteria) prior to delivery. Concurrent chemotherapy consisted of cisplatin administered intravenously at 200 mg/m² per cycle divided over 2–3 cycles (21-day intervals). The control group strictly adhered to the institutional Guidelines for Management of Radiation-Induced Oral Mucositis, encompassing stepped analgesic therapy (from NSAIDs to strong opioids), mucosal repair agents (vitamin B12 spray four times daily), anti-infective treatment (oral fluconazole 150 mg/day for fungal infections; intravenous ornidazole 0.5 g plus levofloxacin 0.5 g once daily for bacterial infections), and nutritional support (enteral nutrition initiated when PG-SGA ≥9). Participants in the experimental arm were supplemented with oral yeast-based β-glucan (Suledox^®^; ≥85% purity) administered daily. The dosage regimen was weight-adjusted, providing 5 grams per 10 kg of body weight, with a maximum daily intake capped at 30 grams. This was divided into three doses dissolved in 200 ml of milk or juice and administered before meals, with ≥1-hour intervals from other oral medications.

### Outcome measures

Mucositis assessors were not blinded to treatment allocation; however, they were not directly involved in the patients’ daily clinical management or treatment decision-making, thereby partially reducing performance bias. Assessments were performed independently using standardized RTOG criteria. For grading discrepancies greater than one level, a senior radiation oncologist, who was not involved in treatment allocation, conducted independent adjudication to minimize subjectivity. Nevertheless, because blinding was not feasible in this Phase II trial, a risk of assessment bias—particularly for mucositis grading—cannot be fully excluded. This grading system ranges from Grade 0 (no symptoms) to Grade IV (hemorrhagic ulceration). Two associate chief radiation oncologists independently evaluated oral mucosal conditions at baseline and within 24 hours after each weekly radiotherapy session using standardized cold light sources and sterile tongue depressors. For grading inconsistencies greater than one level, resolution was achieved through independent evaluation by a senior radiation oncologist. Secondary outcome measures comprised: ① Nutritional evaluation: Weekly in-person PG-SGA evaluations performed by accredited dietitians, where scores of 9 or higher indicated significant nutritional deficiency; ② Body composition analysis: Fasting-state measurements collected using the IQ153 body composition analyzer (SELVAS Healthcare, Inc., Korea), including weight (0.1 kg precision), percent body fat, visceral adipose tissue area (cm²), and skeletal muscle mass (kg), with triplicate measurements averaged for each subject; ③ Laboratory parameters: Fasting venous blood samples collected weekly were analyzed for complete blood count (including leukocytes, hemoglobin, and platelets) using an automated hematology analyzer (Model BC-7500, Shenzhen Mindray Bio-Medical Electronics Co., Ltd., China), while blood cell subsets were quantified by flow cytometry (Model DxH 800, Beckman Coulter, USA).

### Statistical analysis

Given the Phase II exploratory nature of the trial, analyses were interpreted as hypothesis-generating, and the study may have been underpowered for some secondary outcomes. All data processing, statistical computations, and visualization were conducted using SPSS version 25.0 and GraphPad Prism 9.0. The Shapiro-Wilk test evaluated normality assumptions for continuous variables. Data conforming to normal distribution were reported as mean values with (mean ± SD). Independent samples *t*-tests analyzed between-group differences for normally distributed variables, whereas paired *t*-tests examined within-group temporal variations. Multi-group analyses employed one-way ANOVA with Bonferroni-adjusted *post hoc* comparisons.For nonparametric data, results were presented as medians with interquartile ranges [M (IQR)]. The Mann-Whitney U test assessed between-group differences, the Wilcoxon signed-rank test evaluated within-group changes, and the Kruskal-Wallis test compared multiple groups. Categorical variables were summarized as counts with proportions [*n* (%)], analyzed using *χ²* tests, Fisher’s exact tests, or Mann-Whitney U tests for ordinal variables, as dictated by data properties. All analyses used two-tailed tests, with *p*-values below 0.05 considered statistically significant. Given the exploratory Phase II design and absence of a formal *a priori* power calculation, all secondary endpoint analyses (including pain scores, body-composition indices, and immune-cell subsets) should be considered hypothesis-generating. The study may have been underpowered to detect modest differences in these outcomes.

## Results

### Patient enrollment and baseline characteristics

The study initially recruited 63 individuals diagnosed with locoregionally advanced nasopharyngeal carcinoma, who were subsequently randomized into either the experimental or control arm. Three participants discontinued their involvement for personal considerations, resulting in a final cohort of 60 subjects (30 per treatment group) who completed the trial protocol. Demographic and clinical characteristics at baseline showed no statistically significant differences between the two arms (all P-values > 0.05), as detailed in **Table 1**.

**Table 1:** Baseline characteristics of the patient.

Variable	Experimental group (n=30)	Control group (n=30)	P-value
Gender			0.184
Male	9 (30.00%)	14 (46.67%)	
Female	21 (70.00%)	16 (53.33%)	
Age (years)	49.53 ± 12.89	45.03 ± 12.76	0.179
Smoking			0.766
Yes	7 (23.33%)	8 (26.67%)	
No	23 (76.67%)	22 (73.33%)	
BMI	24.66 ± 4.44	22.89 ± 3.14	0.08
Clinical Stage			0.556
II	1 (3.33%)	0 (0%)	
III	16 (53.33%)	15 (50.00%)	
IVa	13 (43.33%)	15 (50.00%)	
Cervical Lymph Node Metastasis			0.085
Yes	25 (83.33%)	29 (96.67%)	
No	5 (16.67%)	1 (3.33%)	

The experimental group exhibited a mean age of 49.53 ± 12.89 years with 30.0% males (n=9). In comparison, control participants showed a younger age distribution (45.03 ± 12.76 years) and higher male representation (46.7%, n=14). Most patients were in clinical stage III. Specifically, in the experimental group, 16 patients (53.33%) were in stage III and 13 (43.33%) in stage IVa. In the control group, 15 patients (50.00%) were in stage III and 15 (50.00%) in stage IVa. In addition, no significant differences were observed between the groups in smoking history, BMI, or cervical lymph node metastasis status (all P > 0.05). Statistical analysis demonstrated comparable baseline characteristics between study arms (all P>0.05), confirming appropriate group homogeneity at trial initiation.

### PGG intervention significantly improves oral mucositis outcomes in NPC patients undergoing chemoradiotherapy

Radiotherapy-induced oral mucositis represents a frequent and clinically significant treatment-related adverse effect in nasopharyngeal carcinoma patients undergoing concurrent chemoradiation, often serving as a major dose-limiting complication. Severe mucositis causes intense pain, dysphagia, and malnutrition, and may necessitate unplanned radiotherapy interruptions. This directly compromises tumor control and impairs quality of life. Thus, effective prevention and management of oral mucositis are crucial for ensuring the successful completion of NPC treatment and improving patients’ quality of life.

The present investigation assessed the therapeutic potential of β-glucan (PGG) supplementation for reducing the severity of radiation-induced oral mucositis in patients undergoing chemoradiation for nasopharyngeal carcinoma. Baseline assessments revealed comparable mucositis grades between the intervention and control arms (P = 0.265), as detailed in **Table 2**.

**Table 2:** Comparison of oral mucositis between groups at different phases.

	Mucositis grade	Experimental group (n=30)	Control group (n=30)	Mann-Whitney U	P-value
Screening Phase				408.000	0.265
	0	0 (0%)	2 (6.67%)		
	1	27 (90.00%)	26 (86.67%)		
	2	3 (10.00%)	2 (6.67%)		
Treatment Phase				266.000	0.004
	0	5 (16.67%)	1 (3.33%)		
	1	16 (53.33%)	10 (30.33%)		
	2	7 (23.33%)	11 (36.67%)		
	3	2 (6.67%)	8 (26.67%)		

Following the intervention, the experimental group demonstrated significantly better outcomes in reducing oral mucositis severity. In the experimental group, 70.00% of patients had mucositis grades 0 - I, compared to 36.67% in the control group. A statistically significant reduction in grade III mucositis incidence was observed in the experimental group (6.7%) versus controls (26.7%) (P<0.05). Importantly, no cases of grade IV mucositis were documented in either treatment arm (**Table 2**).

These results indicate that PGG intervention can significantly reduce severe mucositis incidence and maintain mucosal damage within mild ranges for most patients. These findings carry substantial clinical relevance for maintaining uninterrupted radiotherapy regimens and improving health-related quality of life in affected patients.

### Effect of PGG intervention on pain perception in NPC patients undergoing chemoradiotherapy

Analysis of pain severity scores (**Table 3**) revealed comparable outcomes between treatment arms throughout the 4-week radiation therapy period. Both the experimental group and control group maintained median pain levels of 1–2 at all weekly assessments (weeks 0-4). Intergroup comparisons showed no statistically significant differences at any timepoint (all P>0.05).

**Table 3:** Comparison of pain scores between groups at different time points.

Time (week)	Experimental group (n=30)	Control group (n=30)	P-value
0	1 (1, 1)	2 (2, 2)	0.305
1	1 (1, 2)	2 (1, 2)	0.305
2	1 (1, 2)	2 (1, 2)	0.198
3	1 (1, 2)	2 (1, 2)	0.121
4	1 (1, 2)	2 (1, 2)	0.253

This indicates that while the PGG intervention effectively reduced the severity of mucositis, it did not significantly affect patients’ pain perception during treatment.

Pain assessment data exhibiting non-normal distribution were analyzed using median values with interquartile ranges. The severity grading scale was defined as follows: Grade 1 (mild) = 0–3 points; Grade 2 (moderate) = 4–6 points; Grade 3 (severe) = 7–10 points.

### Effect of PGG intervention on PG-SGA nutritional scores in NPC patients undergoing chemoradiotherapy

Deterioration in nutritional status, as indicated by an increase in PG-SGA scores, is a common complication during chemoradiotherapy for NPC. This decline in nutrition directly impacts treatment tolerance, immune function, and patient quality of life. This investigation assessed the impact of PGG administration on nutritional status parameters. Baseline assessments (Week 0) revealed comparable PG-SGA scores between groups (P = 0.966, **Table 4**). Throughout the initial three weeks of therapy (Weeks 1-3), nutritional status remained statistically similar between cohorts. However, by Week 4, the experimental group demonstrated significantly improved nutritional outcomes (PG-SGA: 10.93 ± 2.60) relative to controls (12.37 ± 2.39; P = 0.030). These findings suggest that while PGG supplementation did not entirely prevent nutritional decline, it significantly mitigated deterioration during advanced treatment phases, offering valuable support for nutritional optimization in chemoradiotherapy patients.

**Table 4:** PG-SGA scores of both groups during screening and treatment phases.

Time (week)	Experimental group (n=30)	Control group (n=30)	T-value	P-value
0	5.80 ± 2.78	5.83 ± 2.64	0.043	0.966
1	8.57 ± 4.22*	8.87 ± 2.62***	0.331	0.742
2	10.03 ± 3.40***	10.53 ± 2.47***	0.652	0.517
3	10.13 ± 2.87***	10.90 ± 2.02***	1.202	0.234
4	10.93 ± 2.60***	12.37 ± 2.39***	2.223	0.030
F-value	11.840	31.440		
P-value	<0.001	<0.001		

Compared with week 0, *P < 0.05, **P < 0.01, ***P < 0.01.

### PGG’s effect on maintaining body composition homeostasis in NPC patients undergoing chemoradiotherapy

During chemoradiotherapy for NPC, body mass index (BMI) is a key indicator of patients’ nutrition and health status. Its stability is crucial for treatment tolerance and recovery.

The present investigation analyzed body mass index (BMI) variations between treatment arms to evaluate the nutritional effects of PGG supplementation. Initial assessments (Weeks 1-2) showed no statistically significant intergroup BMI differences (P = 0.179 and P = 0.226 respectively; **Table 5**). However, subsequent measurements revealed significantly greater BMI values in the experimental group compared to controls at Week 3 (P = 0.026) and Week 4 (P = 0.049). These results demonstrate that PGG administration contributes to BMI preservation and potential improvement during treatment. Short-term changes in BMI and adiposity during chemoradiotherapy may be influenced by transient factors such as hydration status, fluid shifts, corticosteroid exposure, and acute catabolic stress. Therefore, these findings should not be interpreted as definitive evidence of metabolic or nutritional recovery.

**Table 5:** Comparison of changes in BMI between the two groups before and after the intervention.

Time (week)	Experimental group (n=30)	Control group (n=30)	Mann-Whitney U	P-value
1	24.47 ± 4.50	22.75 ± 3.12	358.50 (1007, 823.5)	0.179
2	24.40 ± 4.52	22.70 ± 3.43	367.50 (997.5, 832.5)	0.226
3	24.30 ± 4.48	21.73 ± 3.02	299.50 (1066, 764.5)	0.026
4	23.93 ± 4.68	21.54 ± 2.93	317.00 (1048, 782)	0.049
Kruskal-Wallis statistic	0.841	5.218		
P-value	0.840	0.157		

### PGG’s effect on body fat percentage in NPC patients undergoing chemoradiotherapy

Body fat percentage is a clinically relevant index of body composition and nutritional status and has been associated with energy reserves, immune competence, and treatment tolerance in patients receiving chemoradiotherapy. In the current investigation, we compared longitudinal changes in adiposity measures between treatment arms (**Table 6**). Body fat percentage did not differ significantly between groups at Week 2 (P = 0.597) or Week 4 (P = 0.948). These results suggested that, within the limited treatment window, PGG supplementation did not measurably change overall body fat percentage at the group level. Notably, the experimental group showed numerically higher body fat percentages than controls across time points, although the differences were not statistically significant. This pattern might be consistent with a tendency toward adipose tissue preservation during chemoradiotherapy; however, given the sample size and the exploratory nature of this endpoint, it should be interpreted cautiously. Importantly, body fat percentage reflects only one dimension of nutritional status and may not capture short-term changes in energy balance during intensive treatment. When considered together with the observed benefit of PGG on BMI and other nutritional indicators, these findings raise the possibility that PGG could contribute to maintaining body composition during chemoradiotherapy, but confirmation in larger, adequately powered studies with comprehensive body-composition assessment is warranted.

**Table 6:** Inter-group comparison of body fat percentage changes in NPC patients across intervention phases.

Time (week)	Experimental group (n=30)	Control group (n=30)	T-value	P-value
2	24.11 ± 7.89	25.23 ± 8.04	0.532	0.597
4	24.38 ± 7.93	24.24 ± 8.57	0.066	0.948
T-value	0.789	1.282		
P-value	0.454	0.210		

### PGG intervention and visceral fat area in NPC patients undergoing chemoradiotherapy

Visceral fat area (VFA) is an imaging-derived measure of fat distribution and has been linked to metabolic regulation and systemic inflammation; nevertheless, in oncology settings its clinical interpretation can be context-dependent, particularly during periods of treatment-related anorexia and weight loss. We analyzed longitudinal changes in VFA between treatment arms (**Table 7**). VFA values were higher in the experimental group than in controls at Week 2 (P = 0.010) and Week 4 (P = 0.020). Over time, VFA remained relatively stable in the experimental group (t=0.440, P = 0.663), whereas the control group showed a non-significant upward trend (t=1.458, P = 0.156). These observations may indicate that PGG was associated with preservation of visceral adipose stores during chemoradiotherapy; however, this outcome was secondary/exploratory and was not adjusted for multiple comparisons, and baseline differences or unmeasured confounding cannot be excluded. Importantly, an increase in VFA should not be interpreted as inherently beneficial or as evidence of improved metabolic health, because excess visceral adiposity is generally associated with cardiometabolic risk. In the present clinical context, the finding is best viewed as a potential marker of altered energy balance or nutritional trajectory during intensive treatment. Future studies should validate this association and incorporate mechanistic assessments (e.g., dietary intake, physical activity, insulin resistance markers, and inflammatory profiles) to clarify whether VFA changes reflect nutritional preservation, treatment tolerance, or other metabolic effects.

**Table 7:** Comparison of visceral fat area changes between groups post-intervention.

Time (week)	Experimental group (n=30)	Control group (n=30)	T-value	P-value
2	103.52 ± 51.49	73.80 ± 34.08	2.671	0.010
4	100.23 ± 54.37	71.77 ± 34.10	2.389	0.020
T-value	0.440	1.458		
P-value	0.663	0.156		

### The influence of PGG on the percentage of muscle mass in NPC patients undergoing radiotherapy and chemotherapy

Muscle mass percentage is a critical indicator for assessing body composition and nutritional status, closely associated with patients’ physical strength, metabolic health, and treatment tolerance, making it particularly important for NPC patients. This investigation evaluated longitudinal alterations in muscle mass composition between the experimental group and control group (**Table 8**). Analysis revealed no statistically significant intergroup differences in muscle mass percentage at Week 2 (P = 0.601) or Week 4 (P = 0.962) of the intervention period. This indicates that PGG intervention during chemoradiotherapy did not significantly affect patients’ muscle mass percentage. Nevertheless, combined with its positive impacts on BMI and visceral fat area, these findings suggest that PGG may help patients maintain their overall nutritional status to some extent through multifaceted nutritional support, thereby providing adjunctive support for chemoradiotherapy.

**Table 8:** Compares the percentage changes in muscle mass in each group at the 2nd and 4th weeks of intervention.

Time (week)	Experimental group (n=30)	Control group (n=30)	Mann-Whitney U/T-value	P-value
2	71.17 ± 7.71	69.95 ± 7.75	0.550	0.601
4	70.68 ± 8.47	70.94 ± 8.23	446.50 (918.5, 911.5)	0.962
Wilcoxon/T-value	-138.00(163.5, -301.5)	1.283		
P-value	0.159	0.210		

### Effect of PGG intervention on hematological parameters in NPC patients undergoing chemoradiotherapy

**Figure 1:**
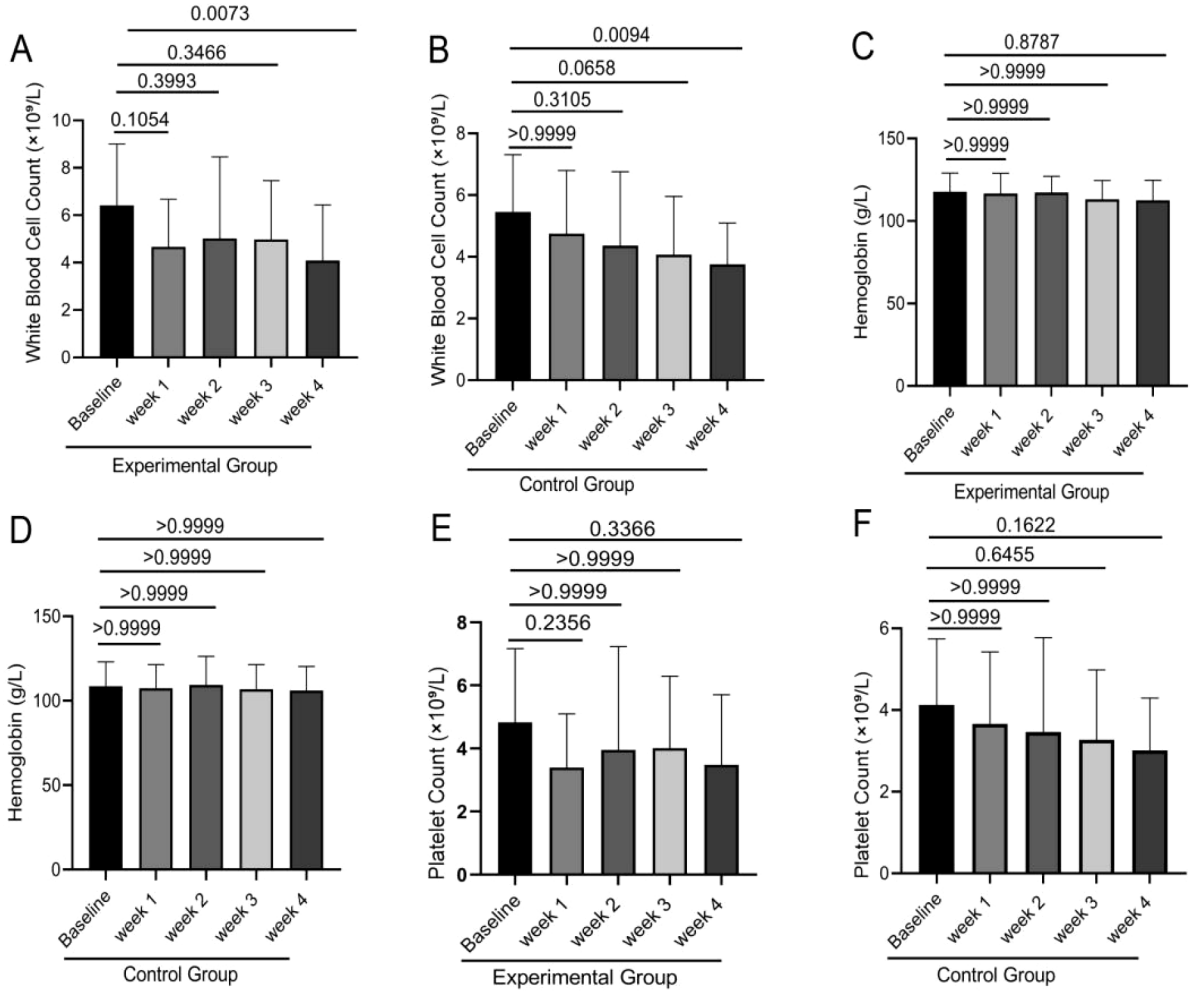
Blood cell counts were determined for both the experimental and control groups at baseline, Week 1, Week 2, Week 3, and Week 4 using an automated hematology analyzer. Intergroup comparisons of white blood cell counts **(A, B)**, hemoglobin levels **(C, D)**, and platelet counts **(E, F)** were performed using one-way ANOVA.

**Figure 2:**
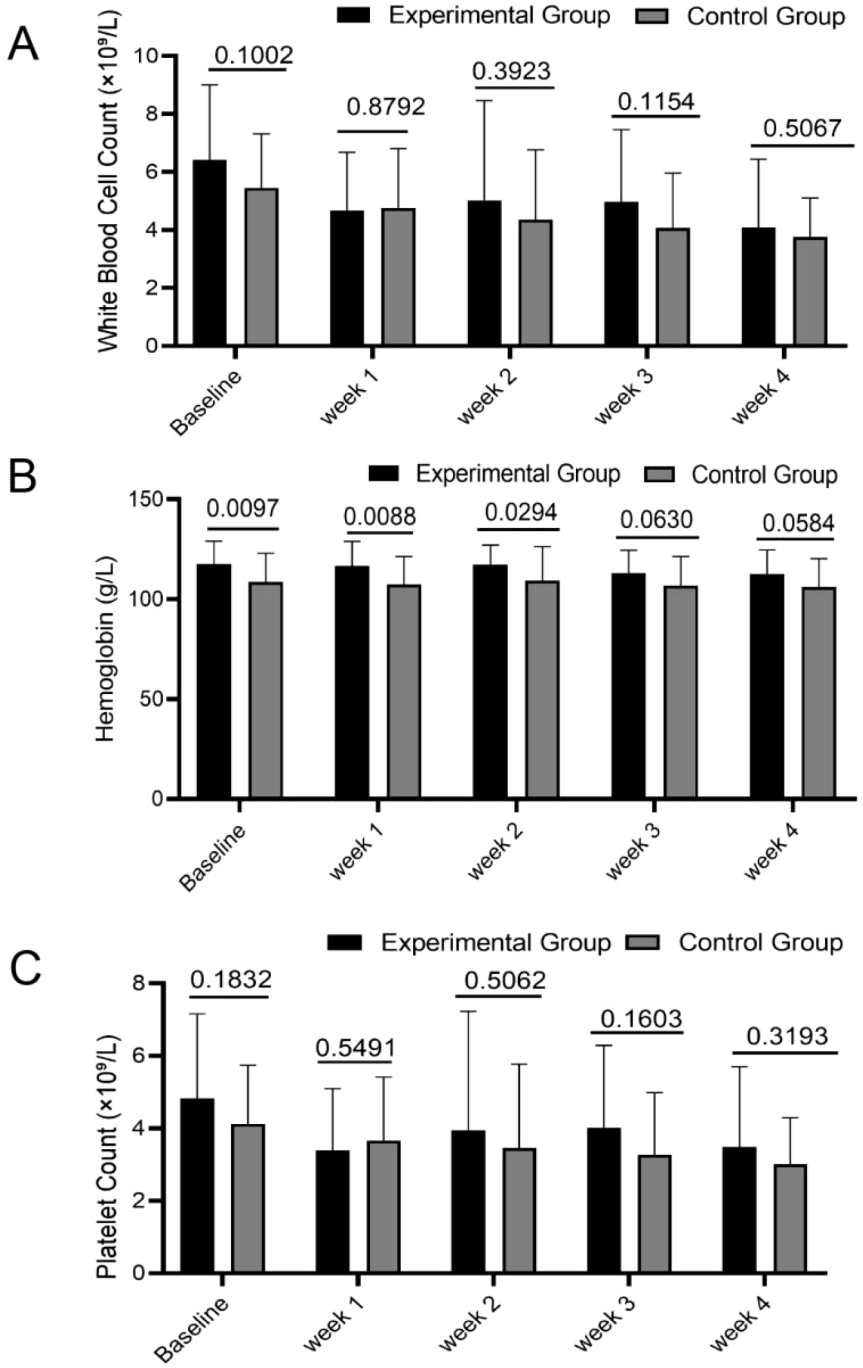
Intra - group comparative analysis of blood cell counts for the experimental and control groups at baseline, Weeks 1, 2, 3, and 4. Paired T - test was used to analyze WBC counts **(A)**, hemoglobin levels **(B)**, and platelet counts **(C)** in both groups.

WBC count, hemoglobin level, and platelet count are critical indicators for evaluating hematopoietic function and treatment tolerance in NPC patients. The stability of these parameters is essential for treatment safety and recovery progression. This study systematically assessed the dynamic effects of PGG intervention on these parameters using **Figures 1** and **2**.

Comparative analysis of hematological parameters revealed no statistically significant alterations from baseline values in either the experimental group or control group during chemoradiotherapy. WBC counts, hemoglobin levels, and platelet counts remained stable across all assessment timepoints (all P>0.05). This indicates that PGG intervention did not significantly affect the overall levels of these hematological parameters.

However, further analysis identified significantly elevated hemoglobin levels in the experimental group relative to controls at baseline, Week 1, and Week 2 (P < 0.05). Throughout the observation period, the experimental group consistently demonstrated higher—though not statistically significant—values for white blood cell counts, hemoglobin, and platelets compared to the control group (all P > 0.05).

These findings suggest that while PGG did not significantly improve hematological parameters, its safety profile was validated with no adverse hematological changes induced by the intervention.

### Effect of PGG intervention on blood cell subset counts in NPC patients undergoing chemoradiotherapy

**Figure 3:**
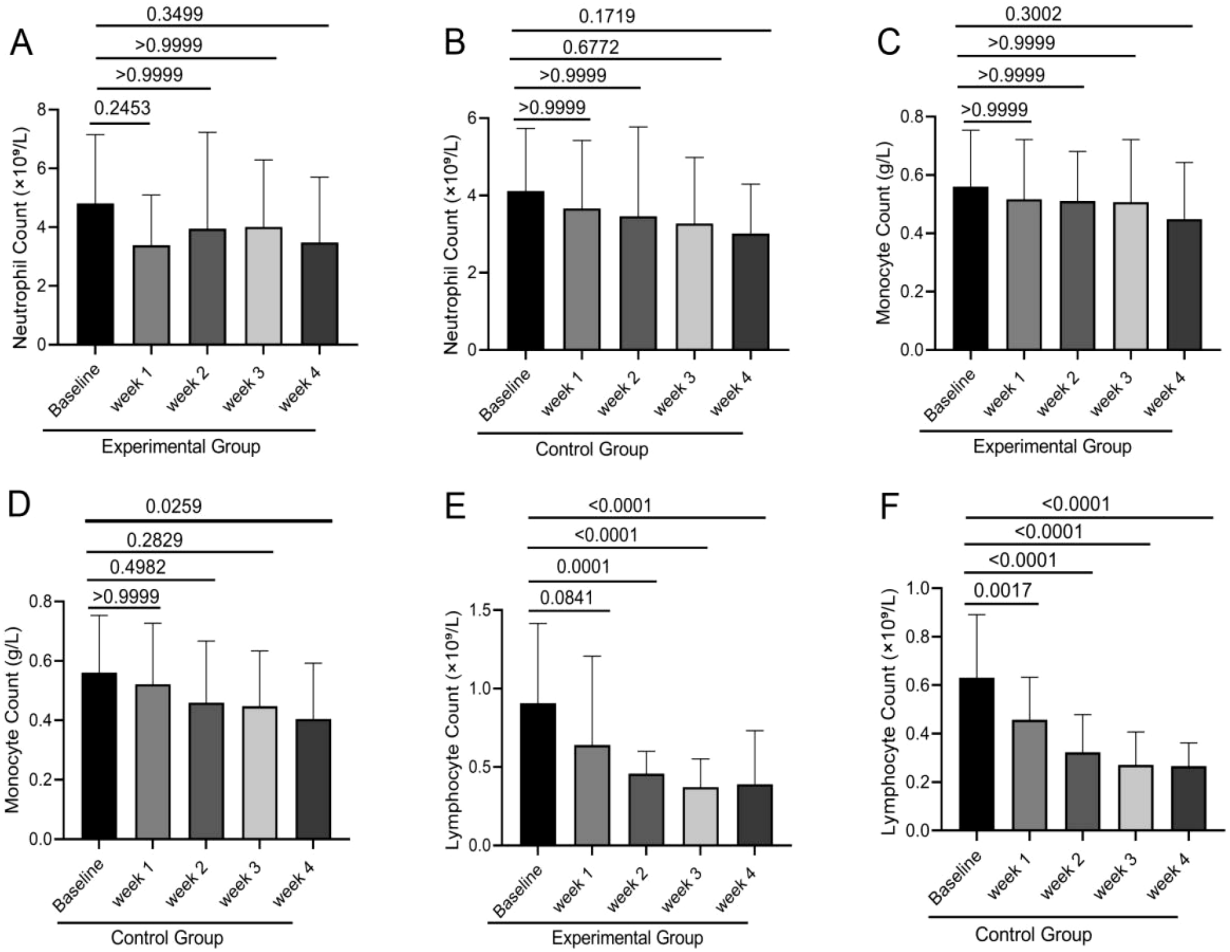
Flow cytometry was performed to analyze blood cell subsets in both the experimental and control groups at baseline, Weeks 1, 2, 3, and 4. Intergroup comparisons of neutrophil counts **(A, B)**, monocyte counts **(C, D)**, and lymphocyte counts **(E, F)** were conducted using one-way ANOVA.

**Figure 4:**
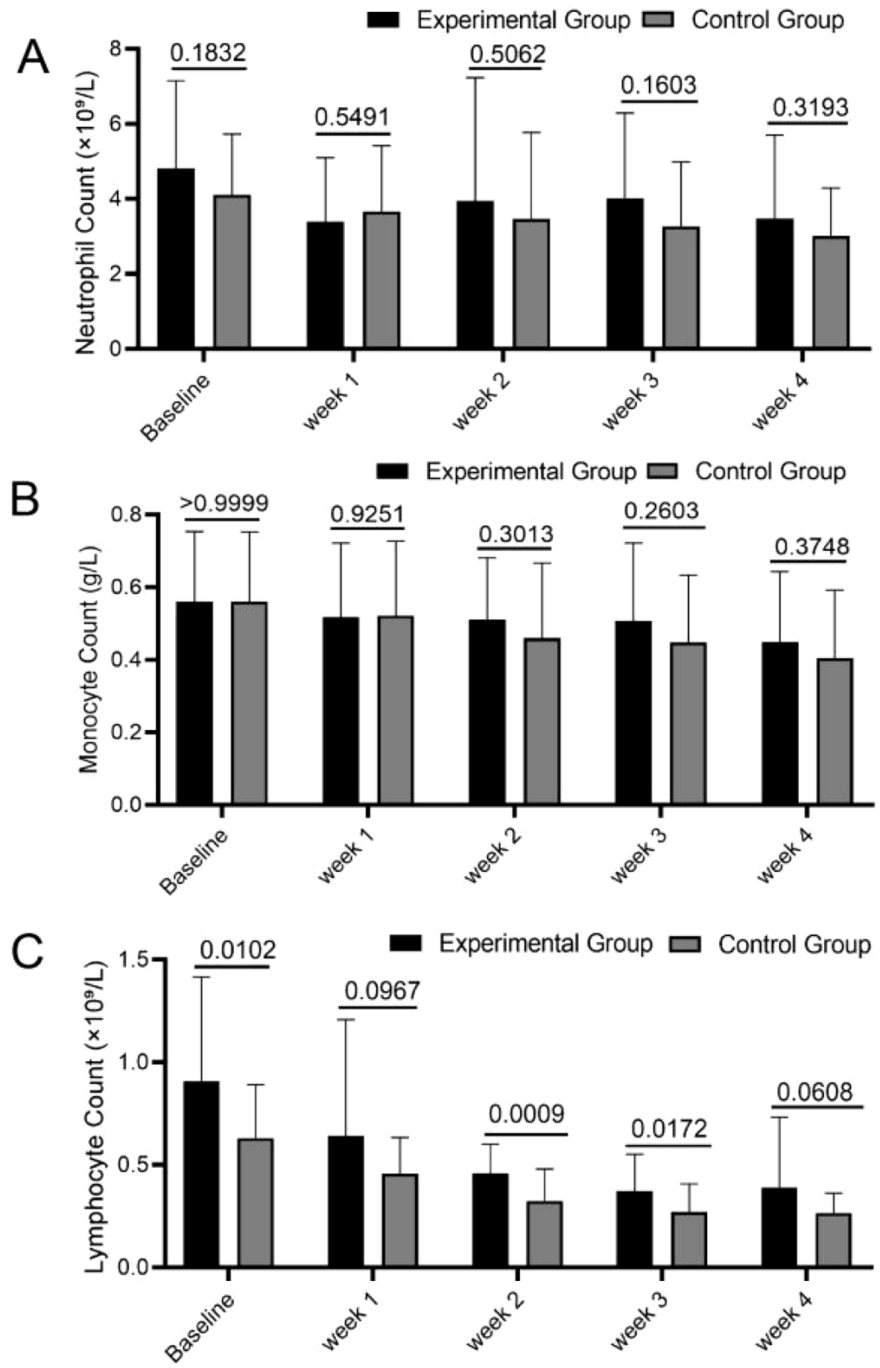
Intragroup comparisons of blood cell subsets within the experimental and control groups across baseline, Weeks 1, 2, 3, and 4 were performed. Paired t-tests were used to analyze neutrophil counts **(A)**, monocyte counts **(B)**, and lymphocyte counts **(C)** for both groups.

Neutrophil count, monocyte count, and lymphocyte count are critical indicators for assessing immune status and hematopoietic function in NPC patients. The stability of these parameters is essential for treatment tolerance and infection risk management. Results demonstrated that in both groups, neutrophil counts and monocyte counts were reduced compared to baseline levels at all time points, though no statistically significant differences were observed (all P > 0.05). Regarding lymphocyte counts, both the PGG and control groups exhibited statistically significant decreases over time compared to baseline (P < 0.05) (**Figure 3**). Comparative evaluation between treatment arms demonstrated consistently greater neutrophil and monocyte counts in the experimental group across all timepoints, though these differences were not statistically significant (all P>0.05) (**Figures 4A, B**). In contrast, lymphocyte counts showed statistically significant elevation in the experimental group compared to controls at baseline and during Weeks 1-3 (P<0.05) (**Figure 4C**). This finding indicates that although PGG intervention did not significantly improve all blood cell subset counts, its potential protective effect on lymphocyte counts may hold clinical value for enhancing patients’ immune function. These immune-cell subset analyses were predefined as secondary endpoints and were exploratory in nature. The trial was not powered to confirm immunomodulatory effects, and therefore any apparent lymphocyte preservation should be interpreted cautiously and requires validation in larger, adequately powered studies.

## Discussion

Studies show that PGG significantly modulates gut microbiota, boosts beneficial bacteria, and regulates intestinal mucosal microecology (21). Additionally, it enhances immunotherapy efficacy by reshaping the tumor microenvironment through increased infiltration and activation of innate and adaptive immune cells (22). These findings provide a theoretical foundation for investigating PGG’s role in mucosal microecology and immune microenvironment regulation. PGG activates the Dectin-1 receptor on macrophages, inducing anti - inflammatory cytokines like IL - 10. It also promotes fibroblast migration and collagen deposition, restoring epithelial barrier integrity (23, 24). Topical PGG application in Zykova’s diabetic foot ulcer study achieved a 40% - 45% wound healing rate (19).

Oral mucositis induced by radiation therapy (RTOM) represents a critical treatment-limiting adverse effect in patients with locoregionally advanced nasopharyngeal carcinoma undergoing concurrent chemoradiation. It stems from radiation - induced DNA damage activating NF - κB - mediated inflammatory cascades. This accelerates apoptosis of epithelial basal cells and delays mucosal repair (25, 26). Despite improvements in locoregional control with cisplatin - based concurrent chemoradiotherapy, it causes high mucositis incidence (78.6% - 88%), with 26.7% - 36.2% progressing to severe (Grade III/IV) mucositis (25, 27, 28). Conventional stepwise management (analgesics, anti - infectives, nutritional support) remains suboptimal for moderate - to - severe mucositis. Toxicity prevents about 40% of patients from completing full - dose chemotherapy, disrupting radiotherapy continuity and survival benefits (29, 30). In this context, our study shows that oral PGG reduces Grade III mucositis incidence by 20 percentage points (6.67% vs. 26.67%, p=0.004) and keeps 70% of patients at Grade 0 - I mucosal injury, surpassing conventional care’s 36.7%.

However, in our study, mucosal lesion improvements were not accompanied by corresponding reductions in pain scores (p>0.05 at all time points). Pain during chemoradiotherapy is multifactorial and may arise from neuropathic sensitization, inflammatory cytokine release, secondary infection, psychological distress, and concurrent analgesic use. Therefore, mucosal healing does not necessarily translate into proportional pain reduction. This dissociation highlights the need for multimodal symptom management strategies beyond mucosal repair alone. This might be due to pain perception being regulated by both neural sensitization and psychological factors. This aligns with the “mucositis - pain dissociation effect” observed by Nutting et al. Their research shows that while β-1, 3/1, 6-glucan mouthwash reduces mucositis severity, pain relief still requires opioids (31). This highlights the need for future combination therapy with neuromodulatory agents (e.g., gabapentin) for comprehensive symptom control. In summary, PGG has significant effects on mucosal microecology and immune microenvironment regulation, supported by its multi - target mucosal repair mechanisms. It also shows great therapeutic potential in diabetic foot ulcers and chemoradiotherapy toxicity models, paving the way for novel clinical applications. In addition, pain may lag behind visible mucosal healing and can be influenced by concurrent infections, neuropathic components, and analgesic use; future studies should incorporate standardized analgesic recording and patient-reported outcome measures to better capture symptom relief.

Regarding nutritional status, PGG supplementation was associated with a modest attenuation of nutritional deterioration during the later treatment phase. At Week 4, the PG-SGA score was lower in the PGG group than in controls (P = 0.030), and BMI was better preserved at Weeks 3–4. Changes in body fat percentage and visceral fat area were evaluated as secondary, exploratory endpoints; while some between-group differences were observed, the clinical implications of short-term body-composition changes during chemoradiotherapy remain uncertain and should not be overinterpreted without confirmation in larger, adequately powered studies with longer follow-up. Importantly, the observed short-term preservation of BMI or visceral fat should not be overinterpreted as proof of sustained nutritional rehabilitation or improved metabolic health. Objective longitudinal assessments, including dietary intake quantification and metabolic biomarkers, are required to confirm true nutritional recovery.

Several methodological constraints should be acknowledged. First, this was a single-center Phase II study with a relatively small sample size, which limited statistical power and generalizability, particularly for secondary and exploratory outcomes. Second, because the intervention could not be fully blinded, mucositis assessors were not blinded to treatment allocation, which may have introduced potential assessment bias despite the use of standardized RTOG criteria and adjudication procedures. Third, no adjustment for multiple comparisons was performed, increasing the risk of type I error for secondary endpoints; minor baseline differences, although not statistically significant, could still have influenced certain exploratory outcomes. Follow-up was limited to the active treatment period. Consequently, the study could not evaluate time to complete mucosal healing, late radiation toxicity, durability of nutritional effects, or oncologic outcomes such as progression-free survival. These limitations restrict direct clinical translation and underscore the need for longer-term follow-up in future trials. Finally, interpretations regarding immune-related indices and body composition parameters (including visceral fat changes) should be considered exploratory and hypothesis-generating, and require validation in larger, multicenter trials. In addition, the lack of observed pain improvement despite reduced mucositis severity warrants further investigation using comprehensive patient-reported outcome measures.

Several methodological constraints should be acknowledged. First, this was a single-center Phase II study with a modest sample size and no formal *a priori* power calculation; therefore, the trial may have been underpowered for several secondary outcomes (e.g., pain scores, body-composition parameters, and immune indices). Because no formal power calculation was performed, negative findings for secondary endpoints—particularly pain scores, body-composition measures, and immune-cell analyses—should not be interpreted as definitive evidence of absence of effect. Second, follow-up was limited to the treatment period, precluding assessment of longer-term mucositis recovery, late toxicities, and oncologic outcomes. Third, mucositis assessors were not blinded to treatment allocation. Although independent grading and adjudication procedures were implemented, the absence of blinding may have introduced assessment bias, particularly for mucositis outcomes, which remain partially subjective despite standardized criteria. Finally, quality-of-life and psychological measures were not systematically collected, which may help explain the lack of observed pain improvement despite reduced mucositis severity.

## Conclusion

In this Phase II randomized trial, oral yeast-derived β-glucan (PGG) reduced the incidence of severe chemoradiotherapy-induced oral mucositis and was associated with better preservation of nutritional status in patients with locoregionally advanced nasopharyngeal carcinoma, without apparent additional toxicity. Findings related to immune-cell subsets should be interpreted as exploratory and hypothesis-generating rather than mechanistically confirmatory. Larger multicenter trials with predefined power calculations, blinded outcome assessment where feasible, and longer follow-up are warranted to confirm efficacy and to define the mechanisms and long-term clinical benefits.

## Data Availability

The raw data supporting the conclusions of this article will be made available by the authors, without undue reservation.
